# Supplementary material for: The mortality cost of carbon
Source: Nat Commun. 2021 Jul 29;12:4467. doi: 10.1038/s41467-021-24487-w (PMC8322393; doi:10.1038/s41467-021-24487-w)
Supplement: Supplementary file 1 — Supplementary Materials [file 41467_2021_24487_MOESM1_ESM.docx]

**The Mortality Cost of Carbon – Supplementary Materials**

**R. Daniel Bressler ^1,2,3^***

**Affiliations:**

^1^ Columbia University School of International and Public Affairs

**^2^** The Earth Institute at Columbia University

^3^ Columbia University Center for Environmental Economics and Policy

*Correspondence to: [rdb2148@columbia.edu](mailto:rdb2148@columbia.edu)

# Systematic Research Synthesis Detailed Methodology^[[1]](#footnote-2)^

To find relevant scientific literature to construct the mortality damage function, we typed the following string into Google Scholar: “climate change AND mortality AND Death AND Global AND Projection.” Because there are a wide variety of disciplines studying the effect of climate change on human mortality, we chose to use Google Scholar to produce results from a wide variety of scholarly literatures.^[[2]](#footnote-3)^ To avoid using outdated studies, we specified that the study had to be published within the last 20 years (September 1999 to the present). This review was conducted in September 2019. This returned a total of 18,800 results. Because Google Scholar sorts its results by relevance, we just considered the first 100 studies.

For each of these 100 studies, we conducted a first pass in which we classified each of the studies according to the following coding:

- 0 – Note related to this analysis (e.g. doesn’t assess human mortality)
- 1 – A strong potential candidate study
- 1tweak – A study that comes up more than once due to tweaking or different versions of the same paper; all papers where multiple versions occur are given this coding
- 2 – Mortality estimates not given
- 3 – Mortality estimates are given, but through a very limited number of health channels
- 4 – Mortality estimate are given, but only in a limited geographic area
- 5 – Mortality estimates are given, but through a limited number of health channels and limited geographically
- 6 – A literature review paper

A histogram of the 100 reviewed studies is shown below:

**Supplementary Figure 1**

A full list of the 100 candidate studies and their coding is given below:

**Supplementary Table 1**

| **Number** | **Title** | **Lead Author** | **Year** | **Coding** |
| --- | --- | --- | --- | --- |
| 1 | [Toward a quantitative estimate of future heat wave mortality under global climate change](https://ehp.niehs.nih.gov/doi/abs/10.1289/ehp.1002430) | [RD Peng](https://scholar.google.com/citations?user=h5wUydwAAAAJ&hl=en&oi=sra) | 2011 | 5 |
| 2 | [Impacts of 21st century climate change on global air pollution-related premature mortality](https://link.springer.com/article/10.1007/s10584-013-0847-8) | [Y Fang](https://scholar.google.com/citations?user=RpW4hYUAAAAJ&hl=en&oi=sra) | 2013 | 3 |
| 3 | [Heat-related mortality risk model for climate change impact projection](https://link.springer.com/article/10.1007/s12199-013-0354-6) | Y Honda | 2014 | 1tweak |
| 4 | [Temperature sensitivity of drought-induced tree mortality portends increased regional die-off under global-change-type drought](https://www.pnas.org/content/106/17/7063.short) | [HD Adams](https://scholar.google.com/citations?user=Q6rrEugAAAAJ&hl=en&oi=sra) | 2009 | 0 |
| 5 | [Global air quality and health co-benefits of mitigating near-term climate change through methane and black carbon emission controls](https://ehp.niehs.nih.gov/doi/abs/10.1289/ehp.1104301) | [SC Anenberg](https://scholar.google.com/citations?user=vNs9dgUAAAAJ&hl=en&oi=sra) | 2012 | 0 |
| 6 | [Projecting future heat-related mortality under climate change scenarios: a systematic review](https://ehp.niehs.nih.gov/doi/abs/10.1289/ehp.1103456) | [C Huang](https://scholar.google.com/citations?user=KYcMNAsAAAAJ&hl=en&oi=sra) | 2011 | 1 |
| 7 | [Global risk of deadly heat](https://www.nature.com/articles/nclimate3322) | [C Mora](https://scholar.google.com/citations?user=PFCmm5MAAAAJ&hl=en&oi=sra) | 2017 | 1 |
| 8 | [Global and regional health effects of future food production under climate change: a modelling study](https://www.sciencedirect.com/science/article/pii/S0140673615011563) | [M Springmann](https://scholar.google.com/citations?user=NZ7drjwAAAAJ&hl=en&oi=sra) | 2016 | 3 |
| 9 | [Projections of seasonal patterns in temperature-related deaths for Manhattan, New York](https://idp.nature.com/authorize/casa?redirect_uri=https://www.nature.com/articles/nclimate1902&casa_token=jopEnolJSY8AAAAA:wo1cnZm9kCQIa48dGfAvT8ILFZLr65V3_xOaCNmD53i5N97MZK8huTk183qxXgUWtwYoynXl6hmeL78Ffg) | T Li | 2013 | 4 |
| 10 | [Impact of climate change on ozone-related mortality and morbidity in Europe](https://erj.ersjournals.com/content/41/2/285.short) | [H Orru](https://scholar.google.com/citations?user=8G5uR6AAAAAJ&hl=en&oi=sra) | 2013 | 5 |
| 11 | [Impact of climate change on ambient ozone level and mortality in southeastern United States](https://www.mdpi.com/1660-4601/7/7/2866) | [HH Chang](https://scholar.google.com/citations?user=5P2MRM0AAAAJ&hl=en&oi=sra) | 2010 | 5 |
| 12 | [Avoided heat-related mortality through climate adaptation strategies in three US cities](https://journals.plos.org/plosone/article?id=10.1371/journal.pone.0100852) | B Stone Jr | 2014 | 5 |
| 13 | [A global overview of drought and heat-induced tree mortality reveals emerging climate change risks for forests](https://www.sciencedirect.com/science/article/pii/S037811270900615X) | [CD Allen](https://scholar.google.com/citations?user=lSzjnAwAAAAJ&hl=en&oi=sra) | 2010 | 0 |
| 14 | [Associations between elevated atmospheric temperature and human mortality: a critical review of the literature](https://link.springer.com/article/10.1007/s10584-008-9441-x) | [SN Gosling](https://scholar.google.com/citations?user=YXt-YDQAAAAJ&hl=en&oi=sra) | 2009 | 1 |
| 15 | [Climate change and heat-related mortality in six cities Part 2: climate model evaluation and projected impacts from changes in the mean and variability of …](https://link.springer.com/article/10.1007/s00484-008-0189-9) | [SN Gosling](https://scholar.google.com/citations?user=YXt-YDQAAAAJ&hl=en&oi=sra) | 2009 | 4 |
| 16 | [Public health impacts of climate change in Washington State: projected mortality risks due to heat events and air pollution](https://link.springer.com/article/10.1007/s10584-010-9852-3) | [JE Jackson](https://scholar.google.com/citations?user=oyMWftgAAAAJ&hl=en&oi=sra) | 2010 | 4 |
| 17 | [Projections of global health outcomes from 2005 to 2060 using the International Futures integrated forecasting model](https://www.scielosp.org/scielo.php?pid=S0042-96862011000700007&script=sci_arttext&tlng=pt) | [BB Hughes](https://scholar.google.com/citations?user=L_gIwFEAAAAJ&hl=en&oi=sra) | 2011 | 3 |
| 18 | [The contribution of outdoor air pollution sources to premature mortality on a global scale](https://www.nature.com/articles/nature15371?platform=oscar&draft=journal) | [J Lelieveld](https://scholar.google.com/citations?user=ia24XqQAAAAJ&hl=en&oi=sra) | 2015 | 0 |
| 19 | [Projections of temperature-related excess mortality under climate change scenarios](https://www.sciencedirect.com/science/article/pii/S2542519617301560) | [A Gasparrini](https://scholar.google.com/citations?user=EmW2GYwAAAAJ&hl=en&oi=sra) | 2017 | 1 |
| 20 | [The potential impacts of climate variability and change on temperature-related morbidity and mortality in the United States.](https://ehp.niehs.nih.gov/doi/abs/10.1289/ehp.109-1240665) | MA McGeehin | 2001 | 6 |
| 21 | [Aging will amplify the heat-related mortality risk under a changing climate: projection for the elderly in Beijing, China](https://www.nature.com/articles/srep28161) | T Li | 2015 | 5 |
| 22 | [Impact of regional climate change on human health](https://www.nature.com/articles/nature04188/) | [JA Patz](https://scholar.google.com/citations?user=uLr2cnMAAAAJ&hl=en&oi=sra) | 2005 | 1 |
| 23 | [Assessing mortality risk from heat stress due to global warming](https://www.tandfonline.com/doi/abs/10.1080/13669870701217375) | K Takahashi | 2007 | 1 |
| 24 | [Variability in temperature-related mortality projections under climate change](https://ehp.niehs.nih.gov/doi/abs/10.1289/ehp.1306954) | T Benmarhnia | 2014 | 1 |
| 25 | [Environment and health: 2. Global climate change and health](http://www.cmaj.ca/content/163/6/729.short) | [A Haines](https://scholar.google.com/citations?user=TJyhU6YAAAAJ&hl=en&oi=sra) | 2000 | 6 |
| 26 | [The interplay of climate change and air pollution on health](https://link.springer.com/article/10.1007/s40572-017-0168-6) | [H Orru](https://scholar.google.com/citations?user=8G5uR6AAAAAJ&hl=en&oi=sra) | 2017 | 0 |
| 27 | [Analysis and valuation of the health and climate change cobenefits of dietary change](https://www.pnas.org/content/113/15/4146?mkt_tok=eyJpIjoiWWpJeU1EVm1ObVE1Tm1NMyIsInQiOiJRTGd0S1JQM1lcL0JQbFVEK2dVUlwvUU52ZHlcLzZFeUYyeEZ0WnpqSDVWSTlcL1g0QXR6MjVlY2U4TmE0WW9CcWpCU2I4eCtsMmNTdXZuQmpRSmhsWGJLdnFRQUVXa1graU5WcUhHeFpEUHFOaFAwUFZ3SXgyTVhGOVg3RXJrMmRyK0cifQ%3D%3D&utm_source=TrendMD&utm_medium=cpc&utm_campaign=Proc_Natl_Acad_Sci_U_S_A_TrendMD_1) | [M Springmann](https://scholar.google.com/citations?user=NZ7drjwAAAAJ&hl=en&oi=sra) | 2016 | 0 |
| 28 | [Projecting heat-related mortality impacts under a changing climate in the New York City region](https://ajph.aphapublications.org/doi/abs/10.2105/AJPH.2006.102947) | K Knowlton | 2007 | 4 |
| 29 | [Climate change effects on human health: projections of temperature-related mortality for the UK during the 2020s, 2050s and 2080s](https://jech.bmj.com/content/68/7/641.short) | S Hajat | 2014 | 4 |
| 30 | [Climate change and human health: impacts, vulnerability, and mitigation](https://www.sciencedirect.com/science/article/pii/S0140673606689332) | [A Haines](https://scholar.google.com/citations?user=TJyhU6YAAAAJ&hl=en&oi=sra) | 2006 | 1tweak |
| 31 | [On the causal link between carbon dioxide and air pollution mortality](https://agupubs.onlinelibrary.wiley.com/doi/abs/10.1029/2007GL031101) | [MZ Jacobson](https://scholar.google.com/citations?user=f0efcBgAAAAJ&hl=en&oi=sra) | 2008 | 0 |
| 32 | [Climate change and human health: impacts, vulnerability and public health](https://www.sciencedirect.com/science/article/pii/S0033350606000059) | [A Haines](https://scholar.google.com/citations?user=TJyhU6YAAAAJ&hl=en&oi=sra) | 2006 | 1tweak |
| 33 | [Comparative risk assessment of the burden of disease from climate change](https://ehp.niehs.nih.gov/doi/abs/10.1289/ehp.8432) | D Campbell-Lendrum | 2006 | 1tweak |
| 34 | [Prevented mortality and greenhouse gas emissions from historical and projected nuclear power](https://pubs.acs.org/doi/abs/10.1021/es3051197) | [PA Kharecha](https://scholar.google.com/citations?user=q-A_ZlcAAAAJ&hl=en&oi=sra) | 2013 | 0 |
| 35 | [Climate change, heat waves, and mortality projections for Chicago](https://www.sciencedirect.com/science/article/pii/S0380133009002275) | [K Hayhoe](https://scholar.google.com/citations?user=LwiZJosAAAAJ&hl=en&oi=sra) | 2010 | 4 |
| 36 | [Global climate change, widening health inequalities, and epidemiology](https://academic.oup.com/ije/article-abstract/35/2/213/694805) | J Sunyer | 2006 | 0 |
| 37 | [Future global mortality from changes in air pollution attributable to climate change](https://www.nature.com/articles/nclimate3354) | [RA Silva](https://scholar.google.com/citations?user=XVmWAbcAAAAJ&hl=en&oi=sra) | 2017 | 3 |
| 38 | [Climate change and human health: present and future risks](https://www.sciencedirect.com/science/article/pii/S0140673606680793) | AJ McMichael | 2006 | 1tweak |
| 39 | [Quantitative risk assessment of the effects of climate change on selected causes of death, 2030s and 2050s](https://apps.who.int/iris/bitstream/handle/10665/134014/9789241507691_eng.pdf) | World Health Organization | 2014 | 1 |
| 40 | [Current and projected heat-related morbidity and mortality in Rhode Island](https://ehp.niehs.nih.gov/doi/abs/10.1289/ehp.1408826) | SL Kingsley | 2016 | 4 |
| 41 | [Valuing the Global Mortality Consequences of Climate Change Accounting for Adaptation Costs and Benefits](https://papers.ssrn.com/sol3/papers.cfm?abstract_id=3224365) | [T Carleton](https://scholar.google.com/citations?user=x9VDHEgAAAAJ&hl=en&oi=sra) | 2019 | 1 |
| 42 | [Climate change and extreme heat events](https://www.sciencedirect.com/science/article/pii/S0749379708006867) | G Luber | 2008 | 6 |
| 43 | [Impact of climate change on heat-related mortality in Jiangsu Province, China](https://www.sciencedirect.com/science/article/pii/S0269749116319303) | [K Chen](https://scholar.google.com/citations?user=jqG8VOwAAAAJ&hl=en&oi=sra) | 2017 | 4 |
| 44 | [Health and climate change: policy responses to protect public health](https://www.thelancet.com/journals/lanet/article/PIIS0140-6736(15)60854-6/fulltext?code\u003dlancet-site) | [N Watts](https://scholar.google.com/citations?user=pY4BcysAAAAJ&hl=en&oi=sra) | 2015 | 1 |
| 45 | [Regional vegetation die-off in response to global-change-type drought](https://www.pnas.org/content/102/42/15144.short) | [DD Breshears](https://scholar.google.com/citations?user=JQOZ4cUAAAAJ&hl=en&oi=sra) | 2005 | 0 |
| 46 | [Global climate change and children's health: threats and strategies for prevention](https://ehp.niehs.nih.gov/doi/abs/10.1289/ehp.1002233) | PE Sheffield | 2011 | 6 |
| 47 | [Dirty-water: estimated deaths from water-related diseases 2000-2020](http://citeseerx.ist.psu.edu/viewdoc/download?doi=10.1.1.452.1699&rep=rep1&type=pdf) | [PH Gleick](https://scholar.google.com/citations?user=ToFRoZ0AAAAJ&hl=en&oi=sra) | 2002 | 0 |
| 48 | [Addressing Global Mortality from Ambient PM2.5](https://pubs.acs.org/doi/abs/10.1021/acs.est.5b01236) | [JS Apte](https://scholar.google.com/citations?user=05tIWeUAAAAJ&hl=en&oi=sra) | 2015 | 0 |
| 49 | [Climate change and future temperature-related mortality in 15 Canadian cities](https://link.springer.com/article/10.1007/s00484-011-0449-y) | [SL Martin](https://scholar.google.com/citations?user=TauRLnIAAAAJ&hl=en&oi=sra) | 2012 | 4 |
| 50 | [Climate-induced forest dieback: an escalating global phenomenon](http://sa.indiaenvironmentportal.org.in/files/Climate-induced%20forest%20dieback.pdf) | [CD Allen](https://scholar.google.com/citations?user=lSzjnAwAAAAJ&hl=en&oi=sra) | 2009 | 0 |
| 51 | [Mortality and greenhouse gas impacts of biomass and petroleum energy futures in Africa](https://science.sciencemag.org/content/308/5718/98.short) | [R Bailis](https://scholar.google.com/citations?user=tyFCUI4AAAAJ&hl=en&oi=sra) | 2005 | 0 |
| 52 | [Projection of future temperature-related mortality due to climate and demographic changes](https://www.sciencedirect.com/science/article/pii/S0160412016302252) | [JY Lee](https://scholar.google.com/citations?user=xySzPukAAAAJ&hl=en&oi=sra) | 2016 | 4 |
| 53 | [Managing the health effects of climate change: lancet and University College London Institute for Global Health Commission](https://www.thelancet.com/journals/lancet/article/PIIS0140-6736(09)60935-1/fulltext) | A Costello | 2009 | 1 |
| 54 | [Food, livestock production, energy, climate change, and health](https://www.sciencedirect.com/science/article/pii/S0140673607612562) | AJ McMichael | 2007 | 0 |
| 55 | [Human health and climate change in Oceania: a risk assessment](https://www.researchgate.net/profile/Penny_Whetton/publication/51986562_Human_health_and_climate_change_in_Oceania_a_risk_assessment_2002/links/00b7d51ba93f07f1df000000.pdf) | AJ McMichael | 2003 | 4 |
| 56 | [Tree die‐off in response to global change‐type drought: mortality insights from a decade of plant water potential measurements](https://esajournals.onlinelibrary.wiley.com/doi/abs/10.1890/080016) | [DD Breshears](https://scholar.google.com/citations?user=JQOZ4cUAAAAJ&hl=en&oi=sra) | 2008 | 0 |
| 57 | [Simultaneously mitigating near-term climate change and improving human health and food security](https://science.sciencemag.org/content/335/6065/183.short) | [D Shindell](https://scholar.google.com/citations?user=MeraHu8AAAAJ&hl=en&oi=sra) | 2012 | 3 |
| 58 | [Projected heat-related mortality in the US urban northeast](https://www.mdpi.com/1660-4601/10/12/6734) | [E Petkova](https://scholar.google.com/citations?user=v4Tc41kAAAAJ&hl=en&oi=sra) | 2013 | 4 |
| 59 | [Projection of heat wave mortality related to climate change in Korea](https://link.springer.com/article/10.1007/s11069-015-1987-0) | [DW Kim](https://scholar.google.com/citations?user=ByUULNQAAAAJ&hl=en&oi=sra) | 2016 | 5 |
| 60 | [Towards more comprehensive projections of urban heat-related mortality: estimates for New York City under multiple population, adaptation, and climate scenarios](https://ehp.niehs.nih.gov/doi/abs/10.1289/EHP166) | [EP Petkova](https://scholar.google.com/citations?user=v4Tc41kAAAAJ&hl=en&oi=sra) | 2014 | 2 |
| 61 | [Public health impact of global heating due to climate change: potential effects on chronic non-communicable diseases](https://link.springer.com/article/10.1007/s00038-009-0090-2) | T Kjellstrom | 2010 | 6 |
| 62 | [Climate change, tropospheric ozone and particulate matter, and health impacts](https://ehp.niehs.nih.gov/doi/abs/10.1289/ehp.11463) | KL Ebi | 2008 | 6 |
| 63 | [Assessing ozone-related health impacts under a changing climate](https://ehp.niehs.nih.gov/doi/abs/10.1289/ehp.7163) | K Knowlton | 2004 | 4 |
| 64 | [Empirical and process-based approaches to climate-induced forest mortality models](https://www.frontiersin.org/articles/10.3389/fpls.2013.00438/full) | [HD Adams](https://scholar.google.com/citations?user=Q6rrEugAAAAJ&hl=en&oi=sra) | 2013 | 0 |
| 65 | [The 2003 heat wave in France: dangerous climate change here and now](https://onlinelibrary.wiley.com/doi/abs/10.1111/j.1539-6924.2005.00694.x) | M Poumadere | 2005 | 2 |
| 66 | [Projections of temperature-attributable premature deaths in 209 US cities using a cluster-based Poisson approach](https://ehjournal.biomedcentral.com/articles/10.1186/s12940-015-0071-2) | [JD Schwartz](https://scholar.google.com/citations?user=7GdrijgAAAAJ&hl=en&oi=sra) | 2015 | 4 |
| 67 | [Global climate change and children's health](https://pediatrics.aappublications.org/content/120/5/e1359.abstract) | KM Shea | 2007 | 6 |
| 68 | [The effect of future ambient air pollution on human premature mortality to 2100 using output from the ACCMIP model ensemble](https://www.atmos-chem-phys.net/16/9847/2016/) | [RA Silva](https://scholar.google.com/citations?user=XVmWAbcAAAAJ&hl=en&oi=sra) | 2015 | 3 |
| 69 | [Projection of temperature-related mortality due to cardiovascular disease in Beijing under different climate change, population, and adaptation scenarios](https://www.sciencedirect.com/science/article/pii/S001393511731770X) | [B Zhang](https://scholar.google.com/citations?user=BRU0rSEAAAAJ&hl=en&oi=sra) | 2018 | 4 |
| 70 | [Long-term projections and acclimatization scenarios of temperature-related mortality in Europe](https://www.nature.com/articles/ncomms1360) | [J Ballester](https://scholar.google.com/citations?user=KFe_s2kAAAAJ&hl=en&oi=sra) | 2011 | 4 |
| 71 | [Co-benefits of mitigating global greenhouse gas emissions for future air quality and human health](https://www.nature.com/articles/nclimate2009) | JJ West | 2013 | 3 |
| 72 | [Erosion of lizard diversity by climate change and altered thermal niches](https://science.sciencemag.org/content/328/5980/894.short) | [B Sinervo](https://scholar.google.com/citations?user=okwuU2kAAAAJ&hl=en&oi=sra) | 2010 | 0 |
| 73 | [Mitigation potential and global health impacts from emissions pricing of food commodities](https://www.nature.com/articles/nclimate3155) | [M Springmann,](https://scholar.google.com/citations?user=NZ7drjwAAAAJ&hl=en&oi=sra) | 2017 | 0 |
| 74 | [Climate change-related health impacts in the Hindu Kush–Himalayas](https://link.springer.com/article/10.1007/s10393-007-0119-z) | KL Ebi | 2007 | 4 |
| 75 | [Influences of climatic and population changes on heat-related mortality in Houston, Texas, USA](https://link.springer.com/article/10.1007/s10584-016-1775-1) | [A Marsha](https://scholar.google.com/citations?user=ZQPuP18AAAAJ&hl=en&oi=sra) | 2016 | 4 |
| 76 | [On underestimation of global vulnerability to tree mortality and forest die‐off from hotter drought in the Anthropocene](https://zslpublications.onlinelibrary.wiley.com/doi/pdf/10.1890/ES15-00203.1) | [CD Allen](https://scholar.google.com/citations?user=lSzjnAwAAAAJ&hl=en&oi=sra) | 2015 | 0 |
| 77 | [The potential impact of climate change on annual and seasonal mortality for three cities in Quebec, Canada](https://ij-healthgeographics.biomedcentral.com/articles/10.1186/1476-072X-7-23) | B Doyon | 2008 | 4 |
| 78 | [Climate change. A global threat to cardiopulmonary health](https://www.atsjournals.org/doi/abs/10.1164/rccm.201310-1924PP) | MB Rice | 2014 | 6 |
| 79 | [Economic risks of climate change: an American prospectus](https://books.google.com/books?hl=en&lr=&id=0QTSBgAAQBAJ&oi=fnd&pg=PR5&dq=climate+change+AND+mortality+AND+Death+AND+Global+AND+Projection&ots=tUikixEaJe&sig=IclNVgfjK_oelOcktLjxvFvHUOU) | T Houser | 2014 | 4 |
| 80 | [Apparent climatically induced increase of tree mortality rates in a temperate forest](https://onlinelibrary.wiley.com/doi/abs/10.1111/j.1461-0248.2007.01080.x) | [PJ Van Mantgem](https://scholar.google.com/citations?user=TFYviMwAAAAJ&hl=en&oi=sra) | 2007 | 0 |
| 81 | [Urban vegetation for reducing heat related mortality](https://www.sciencedirect.com/science/article/pii/S0269749114001882) | [D Chen](https://scholar.google.com/citations?user=9UYD2owAAAAJ&hl=en&oi=sra) | 2014 | 0 |
| 82 | [Mortality trends and setbacks: global convergence or divergence?](https://www.sciencedirect.com/science/article/pii/S0140673604159023) | AJ McMichael | 2004 | 0 |
| 83 | [Global climate change and health: recent findings and future steps](http://www.cmaj.ca/content/172/4/501.short) | RS Kovats | 2005 | 6 |
| 84 | [Projecting future temperature-related mortality in three largest Australian cities](https://www.sciencedirect.com/science/article/pii/S0269749115300889) | [Y Guo](https://scholar.google.com/citations?user=1c9p-KoAAAAJ&hl=en&oi=sra) | 2016 | 4 |
| 85 | [How much disease could climate change cause](https://pdfs.semanticscholar.org/3097/052a0db12a196e173cea63712888386ca5a5.pdf) | DH Campbell-Lendrum | 2003 | 1 |
| 86 | [Global trends in tropical cyclone risk](https://www.nature.com/articles/nclimate1410) | [P Peduzzi](https://scholar.google.com/citations?user=4_iNhG4AAAAJ&hl=en&oi=sra) | 2012 | 0 |
| 87 | [Climate change: challenges and opportunities for global health](https://jamanetwork.com/journals/jama/article-abstract/1909928) | [JA Patz](https://scholar.google.com/citations?user=uLr2cnMAAAAJ&hl=en&oi=sra) | 2014 | 1 |
| 88 | [Climate change, humidity, and mortality in the United States](https://www.sciencedirect.com/science/article/pii/S0095069611001033) | [AI Barreca](https://scholar.google.com/citations?user=Ok6wMVUAAAAJ&hl=en&oi=sra) | 2012 | 4 |
| 89 | [Heat, cold and climate change](https://jech.bmj.com/content/68/7/595.short) | [A Woodward](https://scholar.google.com/citations?user=g9NMWioAAAAJ&hl=en&oi=sra) | 2014 | 6 |
| 90 | [Heatwaves in Vienna: effects on mortality](https://link.springer.com/article/10.1007/s00508-006-0742-7) | HP Hutter | 2007 | 2 |
| 91 | [Economic implications of climate change impacts on human health through undernourishment](https://link.springer.com/article/10.1007/s10584-016-1606-4) | T Hasegawa | 2016 | 3 |
| 92 | [Estimating global impacts from climate change](https://www.sciencedirect.com/science/article/pii/S0959378004000378) | S Hitz | 2004 | 1 |
| 93 | [Temperature and mortality in 11 cities of the eastern United States](https://academic.oup.com/aje/article-abstract/155/1/80/134292) | [FC Curriero](https://scholar.google.com/citations?user=tGUebrsAAAAJ&hl=en&oi=sra) | 2002 | 2 |
| 94 | [Heat, human performance, and occupational health: a key issue for the assessment of global climate change impacts](https://www.annualreviews.org/doi/abs/10.1146/annurev-publhealth-032315-021740) | T Kjellstrom | 2016 | 0 |
| 95 | [Multi-scale predictions of massive conifer mortality due to chronic temperature rise](https://www.nature.com/articles/nclimate2873) | [NG Mcdowell](https://scholar.google.com/citations?user=K4tnghsAAAAJ&hl=en&oi=sra) | 2016 | 0 |
| 96 | Present and potential future contributions of sulfate, black and organic carbon aerosols from China to global air quality, premature mortality and radiative forcing | [E Saikawa](https://scholar.google.com/citations?user=fKKa9dcAAAAJ&hl=en&oi=sra) | 2009 | 0 |
| 97 | [Global trends of fossil fuel reserves and climate change in the 21st century](https://books.google.com/books?hl=en&lr=&id=nPeZDwAAQBAJ&oi=fnd&pg=PA167&dq=climate+change+AND+mortality+AND+Death+AND+Global+AND+Projection&ots=eB3BAnCFHD&sig=CeUzzRUpKPNVtUkqzEeQk3U84MY) | [BR Singh](https://scholar.google.com/citations?user=pBWwqTIAAAAJ&hl=en&oi=sra) | 2012 | 0 |
| 98 | [Attributing human mortality during extreme heat waves to anthropogenic climate change](https://iopscience.iop.org/article/10.1088/1748-9326/11/7/074006/meta) | [D Mitchell](https://scholar.google.com/citations?user=AwFc88UAAAAJ&hl=en&oi=sra) | 2016 | 2 |
| 99 | [Climate change and health in the urban environment: adaptation opportunities in Australian cities](https://journals.sagepub.com/doi/abs/10.1177/1010539510391774) | [HJ Bambrick](https://scholar.google.com/citations?user=wSypy5sAAAAJ&hl=en&oi=sra) | 2011 | 2 |
| 100 | [Climate change and human health: estimating avoidable deaths and disease](https://onlinelibrary.wiley.com/doi/abs/10.1111/j.1539-6924.2005.00688.x) | RS Kovats | 2005 | 1 |

Of the 100 candidate studies, we found 15 studies that were strong potential candidate studies. We then performed a second pass in which we decided which of the studies would be included in the analysis. Ultimately, we decided on 3 studies that sufficiently met the criteria to be included in constructing the mortality damage function: a 2014 WHO Report *Quantitative risk assessment of the effects of climate change on selected causes of death, 2030s and 2050s* (^2^), a 2019 Climate Impact Lab (a collaboration between the University of Chicago, University of California Berkeley, and Rutgers) report *Valuing the Global Mortality Consequences of Climate Change Accounting for Adaptation Costs and Benefits* (^3^), and a Lancet Planetary Health article 2017 *Projections of temperature-related excess mortality under climate change scenarios* (^4^).

The 2014 WHO report projects global excess mortality from a wide variety of channels including undernutrition, malaria, dengue, diarrheal disease, and heat in 2030 and 2050. It accounts for adaptation in the mortality projection from heat, although the effects of adaptation in the undernutrition and disease-related risks appear to be limited. The authors emphasize that despite their efforts to quantify important mortality pathways, their estimates of the future mortality effects of climate change remain incomplete because they could not calculate other pathways including river flooding, water scarcity, and conflict.

The 2019 Climate Impact Lab Report uses an econometric strategy that exploits historical variations in temperatures to find a relationship between mortality and temperature in regions across the globe. They exploit spatial heterogeneities in the mortality-temperature relationship to understand the role that different income levels and demographics play in affecting the climate-mortality relationship. They break the world into 24,378 regions and project incomes, populations, and climate into the future to estimate excess mortality that results from climate change in these regions. Importantly, their approach allows them to account for the benefits of higher incomes and climate adaptations to gain a more accurate estimate of the effect of climate change on mortality accounting for future adaptation. We utilize their reduced-form global projection of the effect of climate change on mortality accounting for adaptation. Their approach allows them to account for climate-mortality effects that are driven by direct changes in the short-run distribution of temperatures such as the net mortality effect of more hot days and fewer cold days, the mortality effect of increased surface ozone formation, and even the effect of hot days on murders and suicides. However, their approach arguably does not fully capture climate-mortality channels that are driven in part by longer-term pathways that are not econometrically identified from shorter-term temperature fluctuations such as some diseases, flooding, and undernutrition.

The 2017 Lancet Planetary Health Report uses a dataset of daily observed mean temperature and mortality counts from locations around the globe from 1984-2015 to estimate temperature-mortality relationships. They project excess mortality for cold and heat and their net change in a number of locations around the globe under RCP 2.6, 4.5, 6.0, and 8.5. Given their statistical strategy, this report has similar limitations to the 2019 Climate impact lab report: climate-mortality effects driven by direct changes in the distribution of temperatures are likely to be captured, but more complex climate-mortality channels such as changes in contagious diseases, flooding, and the effect on food supply are unlikely to be captured. Among the three studies, this study was the most borderline as to whether it would be included. While the study does include projections for a number of locations around the globe, it does not cover all of the world’s population. It covers 9 regions that include all the Americas, Europe, Australia, East Asia, and South East Asia. This represents about 40% of the world’s projected population in 2050 (^5^). Importantly, the study is missing data for regions that are expected to bear the most severe climate change mortality impacts: South Asia, the Middle East, and Africa. To project a global mortality estimate from this report, we used the 2019 UN population prospects projections for the percentage of the world population that is expected to reside in each of the 9 regions used in the report in 2055 and 2095. We then calculated the world population residing in each of the 9 regions as a percentage of the total projected population in each of the 9 regions in 2055 and 2095 so that this percentage for each of the 9 regions adds to 100%. We then multiplied this percentage by the expected percentage increase in the mortality rate in the region given in the report to create a population-weighted global estimate of the increase in the mortality rate. However, this is an underestimate of the global mortality estimate because the original paper leaves out projections for South Asia, the Middle East, and Africa. In addition, unlike the 2019 Climate Impact lab report, this report does not assume adaptation changes. Although this violates one of our idealized criteria, economics literature on climate-mortality adaptation has suggested that in the United States, there has already been significant adaptation to climate change that has ameliorated the mortality effect of hot days, in particular through the adoption of air conditioning (^6^). This has likely already occurred in other rich regions that have widely adopted air conditioning, such as in Europe, much of the Americas, and some countries in East Asia. Much of the expected future benefit of climate-mortality adaptations can be expected to come from emerging countries that adopt air conditioning. The exclusion of the most vulnerable regions contributes towards understating the future global mortality projection while the exclusion of adaptation contributes towards overstating the future global mortality projection. Utilizing the methodology described above, the 2017 Lancet Planetary Health report projects that in RCP 8.5 in 2100, climate change causes a 4.0% increase in the mortality rate. The 2019 Climate Impact Lab Report makes a global projection and accounts for adaptation, and they project that in RCP 8.5 in 2100, climate change causes a 6.6% increase in the mortality rate. Given similarities in the methods of the two reports, this suggests that the net effect of excluding the most vulnerable regions and excluding adaptation may be to understate the risk of mortality.

The 2017 Lancet Planetary Health study still has some shortcomings as discussed above: (1) It does not provide a full global estimate but instead provides estimates for 9 different subcontinental, mostly in higher-income areas, and (2) It did not attempt to account for adaptation. We addressed (1) by using the methodology described above to convert the 2017 Lancet Planetary Health regional estimates into a global estimate. With respect to (2), the economics literature on climate-mortality adaptation has suggested that in the United States, there has already been significant adaptation to climate change that has ameliorated the mortality effect of hot days, in particular through the adoption of air conditioning (^6^). This adaptation has also likely already occurred in other rich regions that have widely adopted air conditioning, such as in Europe, much of the Americas, and some countries in East Asia. Much of the expected future benefit of climate-mortality adaptations can be expected to come from emerging countries (^3^). The exclusion of the most vulnerable regions contributes towards understating the future global mortality projection while the exclusion of adaptation contributes towards overstating the future global mortality projection. Despite these shortcomings, we still decided to use the 2017 Lancet Planetary Health because it appears to be the most global and sophisticated study of the effect of temperature-related excess mortality in the epidemiology literature. It was ultimately a difficult judgement call whether to use the study in our main specification, but with these shortcomings in mind, we ran DICE-EMR with an alternative specification in which the mortality damage function does not include the 2017 Lancet Planetary Health study, shown in supplementary table 4. Excluding the 2017 Lancet Planetary health study increases both the MCC and the SCC. In the DICE baseline emissions scenario, the 2020 SCC increases from $258 to $295 per metric ton. The MCC increases from 2.26x10^-4^ per metric ton (implying that 4,434 metric tons of carbon dioxide released in 2020 -- equivalent to the lifetime emissions of 3.5 average Americans -- causes one excess death globally between 2020-2100) to 2.71x10^-4^ lives per metric ton (implying that 3,690 metric tons of carbon dioxide released in 2020 -- equivalent to the lifetime emissions of 2.9 average Americans -- causes one excess death globally between 2020-2100). In addition, the cumulative number of 2020-2100 excess deaths from climate change increases from 83 million to 95 million.

Of the three studies, only the 2019 Climate Impact Lab study projects lost life-years, although these projections are only alluded to and not provided in the paper. The DICE-EMR mortality damage function measures the increase in the mortality rate as a function of global average temperatures. The MCC captures excess deaths, and not lost life-years; the DICE-EMR SCC treats the welfare loss from an excess death the same regardless of the age of the person dying. This is consistent with the way that cost-benefit analysis is conducted in the United States due to Federal laws against age-discrimination in policy. However, a wider range of future studies are expected to produce projections of lost life-years, and future versions of DICE-EMR may also calculate an MCC that projects lost life-years in addition to excess deaths. Still, it is important for studies that project excess deaths to account for demographic changes even when projecting an increase in the mortality rate due to heterogeneous effects on different age groups (^7^). The 2019 Climate Impact Lab study and the 2014 WHO study account for demographic change in their mortality estimates, while the 2017 Lancet Planetary Health study does not. This is further motivation for the alternative specification that only includes the 2019 Climate Impact Lab study and the 2014 WHO study.

After selecting the studies through the systematic research synthesis described above, we used the studies’ projected increase in the mortality rate under different warming scenarios to construct a dataset that is used for the construction of a reduced-form mortality damage function. For each study, we considered their projections for the increase in the mortality rate in 2030, 2050, 2075, and 2100 for all of the scenarios provided by the study. A summary of this dataset is given below:

**Supplementary Table 2**

| **Study Authors** | **Study Year** | **Year of Impact** | **Region** | **Emissions Scenario** | **Average Temperature (degrees C)** | **Increase in Mortality Rate (Central Estimate)** | **Increase in Mortality Rate (Low Estimate)** | **Increase in Mortality Rate (High Estimate)** | **Analysis Style** | **Mortality Impact as given** | **Notes on Adaptation** | **Survey Notes** |
| --- | --- | --- | --- | --- | --- | --- | --- | --- | --- | --- | --- | --- |
| Carleton et al. | 2018 | 2100 | Global | RCP 8.5 | 4.8 | 6.6% | -2.9% | 17.2% | Statistical | Additional 73 Deaths per 100,000 | Fully accounts for adaptation | Used Magicc 6.0 RCP 8.5 projection for global average temperatures. |
| Carleton et al. | 2018 | 2075 | Global | RCP 8.5 | 3.6 | 2.8% | -2.8% | 8.5% | Statistical | Additional 30 Deaths per 100,000 | Fully accounts for adaptation | Used Magicc 6.0 RCP 8.5 projection for global average temperatures. |
| Carleton et al. | 2018 | 2050 | Global | RCP 8.5 | 2.4 | 0.7% | -2.8% | 4.3% | Statistical | Additional 7 Deaths per 100,000 | Fully accounts for adaptation | Used Magicc 6.0 RCP 8.5 projection for global average temperatures. |
| Hales et al. | 2014 | 2030 | Global | A1B | 1.4 | 0.4% | 0.0% | 0.3% | Enumerative+ Statistical | Total mortality due to undernutrition, Malaria, Dengue, diarrheal disease, and heat in 2050 | Accounts for some adaptations depending on source of mortality | Used Magicc 6.0 A1B projection for global average temperatures. |
| Hales et al. | 2014 | 2050 | Global | A1B | 2.2 | 0.3% | 0.3% | 0.2% | Enumerative+ Statistical | Total mortality due to undernutrition, Malaria, Dengue, diarrheal disease, and heat in 2030 | Accounts for some adaptations depending on source of mortality | Used Magicc 6.0 A1B projection for global average temperatures. |
| Gasparrini et al. | 2017 | 2050-59 | Partially Global | RCP 2.6 | 1.0 | -0.1% | -0.7% | 0.5% | Statistical |  |  | Used Hayhoe et. al 2017 |
| Gasparrini et al. | 2017 | 2090-99 | Partially Global | RCP 2.6 | 1.1 | 0.0% | -0.7% | 0.7% | Statistical |  |  | Used Hayhoe et. al 2017 |
| Gasparrini et al. | 2017 | 2050-59 | Partially Global | RCP 4.5 | 2.1 | 0.0% | -1.0% | 1.1% | Statistical |  |  | Use Magicc 6.0 projection for global average temperatures. |
| Gasparrini et al. | 2017 | 2090-99 | Partially Global. | RCP 4.5 | 2.6 | 0.4% | -1.5% | 2.3% | Statistical |  |  | Use Magicc 6.0 projection for global average temperatures. |
| Gasparrini et al. | 2017 | 2050-59 | Partially Global | RCP 6.0 | 2.0 | -0.1% | -1.0% | 0.9% | Statistical |  |  | Use Magicc 6.0 projection for global average temperatures. |
| Gasparrini et al. | 2017 | 2090-99 | Partially Global | RCP 6.0 | 3.1 | 1.0% | -1.9% | 4.2% | Statistical |  |  | Use Magicc 6.0 projection for global average temperatures. |
| Gasparrini et al. | 2017 | 2050-59 | Partially Global | RCP 8.5 | 2.7 | 0.5% | -1.5% | 2.2% | Statistical |  |  | Use Magicc 6.0 projection for global average temperatures. |
| Gasparrini et al. | 2017 | 2090-99 | Partially Global | RCP 8.5 | 4.6 | 4.0% | -4.4% | 11.0% | Statistical |  |  | Use Magicc 6.0 projection for global average temperatures. |

As each of the three studies used to construct the mortality damage function show (2014 WHO, 2019 Climate Impact Lab, and 2017 Lancet Planetary Health), there are expected to be significant heterogeneities in the mortality effect of increasing temperatures in different locations. In general, places that are currently hotter are expected to tend to have more excess deaths for a given increase in temperatures, and some cold places are expected to have net mortality benefits for a given increase in temperatures. The studies project that excess deaths from climate change in hotter areas are expected to outweigh the fewer deaths in colder areas, and the net global effect is expected to be an increase in excess global temperature-related mortality. DICE-EMR uses the global projections given by these studies to construct the mortality damage function. Even though regional heterogeneities are not explicitly modeled in DICE-EMR given that it is a single region (that single region being the globe) climate-economy model with a single representative agent, these heterogeneities are still accounted for because the studies used to create the mortality damage function make global projections that are net of these heterogeneities. For example, the Climate impact lab report divides the world into 24,378 regions. For each of these 24,378 regions, they estimate the mortality impact of climate change in different climate scenarios accounting for heterogeneities in climate, economic development, expected levels of adaptation, and demographic structure. They find that, due to heterogeneities in these various factors, there are significant differences around the world in terms of the projected impact of climate change on mortality. While they are able to make specific mortality projections for all 24,378 regions, they are also able to project the net global mortality effect accounting for the various heterogeneities in all 24,378 regions that they analyze. Similarly, the 2014 WHO report accounts for heterogeneities between regions and it provides regional projections for 21 different regions. They also provide a global estimate, which is the number of projected excess deaths globally in different scenarios, which is just an aggregation of these regional effects. As with the Climate Impact Lab report, DICE-EMR use the global estimates as inputs to the mortality damage function estimation. The estimates from the 2017 Lancet Planetary Health report are converted into a global projection using the methodology described above. After collecting the projections in supplementary table 2, creating the mortality damage function is simply a curve-fitting exercise to map the global mortality projections made in the scholarly literature to the associated increase in global average temperatures in the scenario in which those projections are made.

In addition, there is an extended module embedded within DICE-EMR that includes estimates of the effect of climate change on intergroup and interpersonal conflict, and then projects the effect of these changes in conflict on the mortality rate using estimates from (^8,9^). However, we ultimately concluded that this literature was too nascent to fully meet the criteria specified in the systematic research synthesis to be included in this study. In particular, it was unclear how to project the likely effects of adaptation since adaptation to avoid intergroup conflicts in the face of climate change is not well-established yet theoretically or empirically. However, as the literature advances, future versions of DICE-EMR may be able to account explicitly for the welfare and mortality costs of climate-related conflict.

# Curve Fitting and Sensitivity Analysis

A scatterplot of the central estimate increase in the mortality rate as a function of the increase in global average temperatures from the studies in Supplementary Table 2 is shown below.

**Supplementary Figure 2**


*Green dots are estimates from Gasparrini et al 2017, purple dots are estimates from Hales et al 2014, blue dots are estimates from Carleton et al 2019. Produced in Stata.*

We estimate the mortality damage function by fitting a curve through this data. We do this by running a weighted regression where each study is given 1/3 weight, and each data point within a study is given proportional weight. We ran this weighted regression using Matlab’s curve fitting tool for a number of functional forms, shown in supplementary figure 3 and supplementary table 3. These include (A) linear, (B) quadratic, (C) 3^rd^ order polynomial, (D) exponential, (E) power, and (F) two-parameter Weibull. As supplementary figure 3 and supplementary table 3 show, the linear curve (A) produces a relatively poor fit while each of the non-linear functional forms (B-F) produce similar curves that provide an excellent fit. To maintain consistency with the functional form of the climate-economy damage function in the original DICE model, we chose to use the quadratic functional form.

In addition to estimating the mortality damage function for the central estimates given by the studies, we also ran two separate quadratic weighted regressions for the high and low estimates using the same methodology as described above to produce mortality damage functions in the high and low scenarios. As with the central estimate, each study is given 1/3 weight, and each data point within a study is given proportional weight. The results of this are shown in figure 6 of the main text. The specific functions for these mortality damage functions are shown in the DICE-EMR model in the data repository (see data availability section), and figure 6 was produced by graphing these functional forms in Wolfram Mathematica.

# Results Excluding Gasparrini et al. 2017

**Supplementary Figure 4**

# Results Excluding Hales et al. 2014

**Supplementary Figure 5**

# Post-21^st^ Century Impacts

The DICE baseline emissions scenario results in an increase in global average temperatures to 4.1° C above pre-industrial by 2100. The warming does not stop there, however, as temperatures continue to increase to a peak of 7.1° C above preindustrial in the 23^rd^ century. Although the studies that we use to construct the mortality damage function only make projections to 2100, and all of these projections are for warming scenarios below 5° C, the mortality damage function that is created by fitting a curve through these projections as described above implies that mortality will continue to increase at an increasing rate through the 6° C and 7° C ranges. The figures below show the projected mortality impacts after 2100. As supplementary figure 6 shows, there are 4.6 million projected yearly excess deaths in 2100, which increases to 18.9 million yearly excess deaths in the mid-23^rd^ century, before falling to 9.6 million by 2500.

Supplementary figure 7 shows cumulative excess deaths in the DICE baseline emissions scenario as well as the DICE-EMR optimized emissions scenario. In the DICE baseline emissions scenario, cumulative excess deaths through 2500 are substantial, totaling 5.610 billion. In the DICE-EMR optimized emissions scenario, we largely avoid the global average temperatures that have an especially damaging effect on human mortality. Global average temperatures reach 2.4° C by 2100 and peak at 2.7° C in the mid-22^nd^ century. Cumulative excess deaths total 85 million by 2500 in the DICE-EMR optimized emissions scenario. Thus, by pursuing the optimized emissions scenario, 98% (5.525 billion out of the 5.610 billion) of the cumulative excess deaths from climate change from 2020-2500 can be avoided.

**Supplementary Figure 6**

**Supplementary Figure 7**

# Derivation of the DICE-EMR Critical Level Isoelastic Utility Function

In order to calculate the SCC and the optimal emissions^[[3]](#footnote-4)^ trajectory^[[4]](#footnote-5)^ in a general equilibrium setting with endogenous mortality, we leverage the methodology developed by Robert Hall & Charles I. Jones to calibrate consumption-equivalent welfare loss from excess deaths in a representative agent general equilibrium macroeconomic model with endogenous mortality (^10^). The derivation below closely follows the similar methodology described by Charles I. Jones in his 2016 Journal of Political Economy Paper *Life and Growth* (^11^).

When evaluating policies that affect life and death in specifications involving per period utility $u\left( c_{t} \right)$, the level of the utility function matters a great deal (^12^). We use the following general utility function:

$$\begin{aligned} u\left( c_{t} \right)=\frac{{c_{t}}^{1-\eta}}{1-\eta}+\bar{u}\#\left( S1 \right) \end{aligned}$$

$\bar{u}$ is an upper bound on utility when $\eta>1$. Following standard practice in the literature, we normalize the utility of death to 0. Equation (S1) can then be interpreted as a critical level utility function (^13,14^), where $\bar{u}=-\frac{\bar{c}^{1-\eta}}{\eta-1}$ and $\bar{c}$ represents the critical level of consumption where the agent is indifferent between life and death:

$$u\left( c_{t} \right)=\frac{{c_{t}}^{1-\eta}}{1-\eta}-\frac{\bar{c}^{1-\eta}}{1-\eta}$$

Thus, when $c_{t}=\bar{c}$ the agent is indifferent between life and death. To calibrate $\bar{c}$, we leverage the calibration method discussed in Jones 2016 (^11^). The term$u\left( c_{t} \right)$ represents the value of life in year t in utils. Dividing by $u'\left( c_{t} \right)$ converts this value into consumption units, so $u\left( c_{t} \right)/u'\left( c_{t} \right)$ represents the value of life in year t in consumption units. Dividing this term by $c_{t}$ then gives the value of life in year t as ratio of the level of consumption in year t: $\frac{u\left( c_{t} \right)/u'\left( c_{t} \right)}{c_{t}}$. We then calculate this figure as a function of $\bar{u}$ and $\eta$ from equation (S2):

$$\begin{aligned} \frac{u\left( c_{t} \right)/u'\left( c_{t} \right)}{c_{t}}=\bar{u}{c_{t}}^{\eta-1}+\frac{1}{1-\eta}\#\left( S2 \right) \end{aligned}$$

Following Jones 2016 (^11^), we can then calibrate the value of life to the value of a statistical life year (VSLY). There is a wide variance in estimates for VSLY (^15^). For instance, the 2019 Climate Impact Lab study that we use in this analysis calculates a partial mortality social cost of carbon in their main specification by using United States EPA VSL (value of statistical life) estimates and converting this to a VSLY estimate (see their appendix H for their methodology). Using their methodology yields a VSLY that is 7.9x the value of per capita consumption (^3^). They also run an alternative specification where they use the VSL estimate from the Ashenfelter & Greenstone 2004 Journal of Political Economy study instead of US EPA estimate to calculate the implied value per life year. With this specification, this yields a VSLY that is 2.8x consumption (^16^). The Charles I. Jones 2016 Journal of Political Economy paper *Life and Growth* (whose methodology we leverage here) uses a central VSLY consumption multiple of 3.5.

Given this range of estimates, we use VSLY estimates of 2x consumption, 4x consumption (our main specification), and 8x consumption. While estimating the VSLY is necessary to calculate the SCC and optimal climate policy, the wide variety of estimates for VSLY is a further motivating reason for the MCC metric that we introduce in this paper that does not attempt to value lives.

Using our main specification, we calculate that $\frac{u\left( c_{t} \right)/u'\left( c_{t} \right)}{c_{t}}=4$. Given that DICE-2016 assumes that $\eta=1.45$, we can then solve for the critical level of utility $\bar{u}$ in the central estimate:

$$4=\bar{u}{c_{t}}^{1.45-1}+\frac{1}{1-1.45}$$

Given that 2020 average world consumption in DICE-EMR is $11.86 thousand, we can solve this equation to find that $\bar{u}=2.04$. Solving for the critical level of consumption $\bar{c}$ given that $\bar{u}=-\frac{\bar{c}^{1-\eta}}{1-\eta}=\frac{\bar{c}^{1-\eta}}{\eta-1}$, we find that $\bar{c}=1.20$. This calibration gives the following utility function used in DICE-EMR:

$$\begin{aligned} u\left( c_{t} \right)=\frac{{c_{t}}^{1-\eta}}{1-\eta}+2.04\#\left( S3 \right) \end{aligned}$$

We also run alternative VSLY calibrations, for instance 2x consumption and 8x consumption. The 2020 value of a life as a multiple of consumption is a tweakable parameter in DICE-EMR, and DICE-EMR automatically updates the utility function calibrations when the parameter is changed. In the main SCC results in table 2, we show results with alternative VSLY assumptions.

DICE-2016 is a single representative agent macroeconomic model, and DICE-EMR keeps this structure while determining the welfare impact of loss in life in a single representative agent general equilibrium setting. This has an important implication: it gives equal weight to deaths no matter where they occur in the world. Alternative methodologies give greater weight to richer individuals that die compared to poorer individuals based on their willingness to pay to avoid death. Since richer individuals have more financial resources, they have a higher willingness to pay to avoid death. The implication of these alternative methodologies is that deaths in richer countries (e.g. in Western Europe, North America) are weighed more than deaths in poorer countries (e.g. in Africa, South Asia). This has a significant effect on the SCC because most of the deaths are projected to be in poorer countries. The IPCC states that the approach taken by DICE-EMR – valuing all lives at the same level – is nearer the truth than the alternative approach of assigning valuing lives based on willingness to pay to avoid death (^17^). Philosopher John Broome lays out this case in more detail (^18^). He argues that an approach that values lives based on willingness to pay to avoid death is mistaken. He argues that lives should be worth and counted the same no matter where they are in the world and no matter how rich the people dying.

# Social Cost of Carbon (SCC) Derivation

The 2020 SCC is determined by the following equations. See figure 5 for variable names and explanations, the supplementary materials for a more detailed explanation, and (^19^) for a full description:

$$\begin{aligned} SCC\left( 2020 \right)=\frac{\partial W}{\partial E\left( 2020 \right)}/\frac{\partial W}{\partial C\left( 2020 \right)} \end{aligned}$$

In DICE-2016,$L_{t}$ and $R_{t}$ are exogenous. Focusing on the damage term (the SCC numerator):

$$\frac{\partial W}{\partial E\left( 2020 \right)}=\frac{\partial\sum_{t=2020}^{t=2510} u(c_{t})L_{t}R_{t}}{\partial E\left( 2020 \right)}$$

This is equivalent to the discounted marginal effect of carbon emissions in every period, and then applying the chain rule:

$$\sum_{t=2020}^{t=2510} \frac{\partial u(c_{t})}{\partial E\left( 2020 \right)}L_{t}R_{t}=\sum_{t=2020}^{t=2510} \frac{\partial u(c_{t})}{\partial c_{t}}\frac{\partial c_{t}}{\partial E\left( 2020 \right)}L_{t}R_{t}$$

In DICE-EMR, $L_{t}$is now affected by emissions. Focusing on the SCC numerator:

$$\frac{\partial W}{\partial E\left( 2020 \right)}=\frac{\partial\sum_{t=2020}^{t=2510} u(c_{t})L_{t}R_{t}}{\partial E\left( 2020 \right)}$$

Applying the product rule, this is equivalent to:

$$\frac{\partial W}{\partial E\left( 2020 \right)}=\sum_{t=2020}^{t=2510} [\frac{\partial u\left( c_{t} \right)}{\partial E\left( 2020 \right)}L_{t}R_{t}+\frac{\partial L_{t}}{\partial E\left( 2020 \right)}u\left( c_{t} \right)R_{t}]$$

Applying the chain rule, this is equivalent to:

$$\frac{\partial W}{\partial E\left( 2020 \right)}=\sum_{t=2020}^{t=2510} [\frac{\partial u\left( c_{t} \right)}{\partial c_{t}}\frac{\partial c_{t}}{\partial E\left( 2020 \right)}L_{t}R_{t}+\frac{\partial L_{t}}{\partial E\left( 2020 \right)}u\left( c_{t} \right)R_{t}]$$

# The Opportunity Cost of Life Methodology

Equations (S1) – (S3) show how the utility function is calibrated to VSLY, and how this then implies a critical level of consumption above which bringing in a life would be a net positive in the social welfare function. How positive this life lived would be is determined by the level of consumption and the $\eta$ parameter, which determines the amount of utility that the agent gets when consuming above this level.

This methodology is designed to work with the population term in the SWF so that lives above this critical level that are not lived due to climate change – whether because they died or because they were not brought into existence in the first place (what John Broome calls *absences*; see *Climate Matters* 2012, chapters 9 and 10) are counted as a welfare loss in the SWF. This can be thought of as an *opportunity cost of life* methodology. Higher mortality leads to lower total social welfare from the opportunity cost of those who could have been alive to enjoy their utility if they or their ancestors had not died as a result of climate-induced mortality.

The critical level utilitarian approach taken by DICE-EMR includes both the welfare loss from direct deaths and absences and both of these are valued at the opportunity cost of the life that could have been lived if climate change did not alter the human population through its effect on mortality. An alternative approach to the opportunity cost of life approach would be to calculate the welfare loss from excess climate deaths based on the VSL. This would account for the welfare-loss from death, but not for the welfare-loss from absences. There is a good deal of argument and discussion in the population ethics literature about how to treat absences. Derek Parfit in *Reasons and Persons* argues for counting absences, especially in scenarios with significant mortality (^20^). John Broome also leans towards this approach, see *Climate Matters* p. 89 (^18^). However, this approach of counting absences goes against an intuition that a lot of people seem to have: what John Broome calls the *Intuition of Neutrality*, see Climate Matters p. 83, though Broome argues that the Intuition of Neutrality is false. Also, the inclusion of absences in an SWF is a primary motivation for those that are working on preventing existential risks because of the large value of the future that may be lost, see e.g. Toby Ord *The Precipice* (^21^) and Nick Bostrom *Existential Risk Prevention as Global Priority* (^22^).

Functionally within the DICE model, however, the magnitude of absences is small relative to deaths in the near-term centuries that are given the most weight in the DICE SWF due to discounting. In the 21^st^ century in the baseline emissions scenario, the ratio of deaths to absences is 5:1. By the end of the 22^nd^ century, the ratio of deaths to absences is 2:1. By the end of the 23^rd^ century, absences finally catch up to deaths and the ratio is 1:1. However, because DICE discounts utility at an annual rate of 1.5%, a util at the end of the 23^rd^ century is worth 1.4% the value of a present-period util in the SWF. One issue with the approach that includes absences is that the age of people dying is important because if deaths are more skewed towards people that are not of reproductive age (the old and young), then there would be fewer absences than we would expect if deaths are distributed uniformly. As discussed in the main text, we are currently limited by the availability of age-specific mortality projections in the literature to construct the DICE-EMR mortality damage function. However, more projections of this sort are expected to be produced soon, so this is something that can be added in future work.

Population levels affect the SCC in the DICE-EMR framework similarly to the ways describe in Scovronick et. al 2017 (^23^). The SCC tends to be higher with larger population because more people would be harmed by the marginal emission, both in terms of people harmed by lost consumption and a larger number of people that would die/be absent due to a marginally higher mortality rate in future years due to the marginally higher level of warming from the marginal emission.

These factors would also affect how the SCC in the opportunity cost of life framework would vary relative to the alternative framework of calculating the welfare loss from excess climate deaths based on the VSL. There would be two competing effects: (1) Because the alternative methodology has exogenous population, the SCC would tend to be more damaging in DICE-EMR because the population is larger for the reasons mentioned above, and (2) Because both deaths and absences are accounted for as welfare loss, this would tend to make marginal emissions more damaging for a given population level. Which of these effects ends up being larger depends on a number of factors including the emissions scenario and subsequent level of warming and subsequent number of climate-related deaths, discounting (higher discounting implies absences are less important since they only accumulate at large levels further in the future), VSL and VSLY assumptions, and more. It would be an interesting and useful exercise to compare the two approaches, as they are both reasonable approaches to population ethics. However, we leave this to future work.

# Mortality Cost of Carbon (MCC) Derivation

The MCC assesses the marginal mortality effect of carbon emissions in units of human lives. It represents the number of excess deaths over a period of time from one ton of carbon-equivalent emissions:

$$\begin{aligned} MCC\left( 2020 \right)=\frac{\partial Aggregate Deaths\left( 2020 to 2100 \right)}{\partial E_{2020}}=\sum_{t=2020}^{t=2100} \frac{\partial L_{t}d_{t}\left[ 1+\delta\left( T_{t} \right) \right]}{\partial E_{2020}} \end{aligned}$$

Where $L_{t}d_{t}\left[ 1+\delta\left( T_{t} \right) \right]$ is the number of excess deaths in each time period after accounting for the climate-mortality effect $\delta\left( T_{t} \right)$. Partially differentiating this term with respect to emissions:

$$\sum_{t=2020}^{t=2100} \frac{\partial\delta\left( T_{t} \right)}{\partial E_{2020}}{L_{t}d}_{t}$$

Applying the chain rule:

$$\begin{aligned} MC\left( 2020 \right)=\sum_{t=2020}^{t=2100} \frac{\partial\delta\left( T_{t} \right)}{\partial T_{t}}\frac{\partial T_{t}}{\partial E_{2020}}{L_{t}d}_{t} \end{aligned}$$

# Discounting Sensitivities

Supplementary figure 8 below shows sensitivities to alternative discounting assumptions. As has been widely discussed in past literature, the SCC is highly sensitive to discounting since damages occur over long timescales, and future damages are converted into present value (^24–27^). In the DICE model, the key discounting parameters are $\eta$ and $\rho$. $\eta$ is a parameter in the utility function described above, and in the context of the DICE model, it is best interpreted as intergenerational inequality aversion (^28^), where a larger $\eta$ implies more aversion to intergenerational inequality. If future generations are expected to be richer, then a smaller $\eta$ would give them greater weight because there is less aversion to the inequality between them and poorer current generations. $\rho$ is the rate of social time preference, and it determines the rate at which future utility is converted into present value. Higher $\rho$ puts more weight on the utility of future generations. In the main specification of the DICE model, Nordhaus assumes that $\eta=1.45$ and $\rho=1.5\%$. In all of the results we present in the main text, we adopt Nordhaus’s main discounting specification. Note that under lower discounting rates in DICE-EMR, the contribution of absences towards lost welfare in the future as discussed above.

**Supplementary Figure 8**

# Additional Macroeconomic Results

In DICE-EMR, climate change affects output from two sources (see figure 5 in the main text): (1) the original DICE-2016 climate-economy damage function that represents the portion of economic output lost due to climate change and (2) reduction in the size of the labor force under warming.

The figure below shows the relative contribution of these two factors in output loss in the DICE baseline scenario. It shows that the contribution of factor (2) is relatively small, increasing from near 0% to 15% by 2100.

**Supplementary Figure 9**

# Mortality Impacts in DICE-2016

Nordhaus and Moffat estimated the climate-economy damage function by surveying the climate impacts literature and selecting 26 studies that made projections of economic damages. They then fit a quadratic curve through these projections using a median-weighted regression (^1^). However, most of these 26 studies were heavily de-weighted because they were either superseded by later studies that were also included or they were determined to have poor methods. Of the 26 studies used in the DICE-2016 survey, only 5 received full or nearly full weight (^29–33^). We reviewed the five highly weighted studies below to determine the extent to which they included mortality damage. Of the five studies, three (^29,31,32^) did not include mortality damages. (^33^) accounted for mortality damages from some climate-exacerbated infectious diseases, but health costs only represent 6% of total damages (and likely included some non-mortality health damages as well). (^30^) included some mortality damages, but human life costs represent 9-10% of the total climate damage at both 2.5° C and 10° C. Since these studies were highly weighted, they have the largest bearing on the DICE climate-economy damage function. Provided that they are somewhat representative of the rest of the studies used in the Nordhaus and Moffat survey, we conclude that mortality damages represent less than 5% of total climate damages in DICE.

Some of the heavily weighted studies include cost estimates for plausible defensive adaptations that could be undertaken to reduce the impact of climate change on mortality. These include higher healthcare expenditures (^29,31^) and higher expenditure on air conditioning (^30^). Since these are market expenditures, they may also be included in studies that determine market impacts to GDP such as (^32^), though they are not mentioned explicitly, and the degree to which they are included is unclear. In addition, the studies that are over 20 years old (^30,33^) were published before comprehensive studies of the health impacts of global warming were available (^33^). For these reasons, we conclude that the costs of defensive adaptation to reduce the mortality impact of climate change in DICE-2016 are likely understated. Although DICE-EMR does not model the costs of defensive adaptation to climate-mortality impacts, this information might be useful for future studies that attempt to explicitly model the costs of adaptation and want to know the extent to which these costs are already included in the DICE-2016 climate-economy damage function.

Dellink et al. 2014^[[5]](#footnote-6)^

Accounts for some changes in morbidity, worker productivity, and demand for healthcare. Mortality costs are not included.

*“Changes in regional labour productivity are considered as the primary channel to account for health impacts. Lower mortality translates in an increased labour productivity which is one-on-one proportional to the change in the total population. The underlying assumption is that health impacts affect the active population, disregarding the age characteristic of cardiovascular and respiratory diseases. This information is complemented with changes in health expenditures, reflecting a need for households and governments to allocate increasing parts of their budget to health.”*

Health impacts make up a small proportion of total GDP impacts as shown in their figure 3.

Bosello et. al 2012

Health effects are only included for the European Union, and only addresses thermal discomfort in “on the job performance.” Mortality costs are not included:

*‘When implemented, the climate change impacts summarized…imply that in 2050, there will be a worldwide GDP loss of -0.5%... This is mainly driven by decreases in crop productivity, followed by the redistribution of tourism flows and land loss to sea-level rise. Other impacts are negligible; however, it is worth recalling that flooding and health in particular are computed for the EU only. In addition, “health”, only addresses thermal discomfort on “on the job” performance.’*

Their figure 2 shows that health is a small fraction of the total damages.

Nordhaus 2006

Only accounts for market impacts to GDP. Mortality impacts not mentioned.

Nordhaus and Boyer 2000

Only accounts for mortality costs from the increase in climate-related infectious diseases due to climate change. They project only this form of health damage because at the time the book was written, they say that “There are currently no comprehensive studies of the health impacts of global warming.” They project health costs to be only 6% of the total cost of climate change.

Cline 1992

At the 2.5° C climate damage estimate, “human life” makes up 9.4% of the total damage. At the 10° C climate damage estimate, human life makes up 9.8% of the total damage. The author notes that at the time of publication, the literature on the effect of climate on health and mortality is very underdeveloped.

It should also be noted that Cline, 1992 provides a US-specific estimate of climate damages even though this study is being used to make a global damage projection in the DICE model.

# Detail on UN Population Projection Methodology

The baseline population projections from the UN are given in the figures below:

**Supplementary Figure 10**

The birth rate is projected to fall significantly over the 21^st^ century due to the continued effects of the demographic transition, especially in developing countries. The mortality rate rises slightly due to an aging population, especially in developing countries.

The 2019 UN population prospects are largely projections of past trends, and the likely future mortality effects of climate change are not factored in. They use probabilistic projections of fertility and mortality rates on a country-by-country basis to derive population projections by 2095.

The demographic transition theory is the basis for projections of future country-specific fertility levels. Less developed countries exhibit high fertility rates before transitioning to a lower fertility rate as the country develops. The fertility projections are informed by historical trends and assume that the conditions facilitating fertility decline will persist in the future.

Assumptions for the projection of mortality are specified in terms of life expectancy at birth. Mortality rates are projected based on development levels. Poor developing countries exhibit some albeit slow growth in life expectancy due to the diffusion of improved hygiene and nutrition. This is followed by a period of accelerated improvements in life expectancy driven mainly by improvements in the mortality of infants and children, especially due to interventions against infectious diseases that often strike in childhood. This period is accompanied by social and economic development along with interventions in public health and basic medical care. As countries continue to develop, life expectancy improves at a slower rate. The easiest gains, mainly against infectious diseases that often strike in childhood, have already been achieved. Countries in this stage mainly improve life expectancy by preventing deaths from non-communicable diseases that more often affect the elderly. These interventions have a lower payoff in years of life expectancy gained from saving an older person compared to saving a child. See (^34^) methodology section for more detail.

Supplementary References:

1. Nordhaus, W. D. & Moffat, A. *A survey of global impacts of climate change: Replication, survey methods, and a statistical analysis*. (2017).

2. Hales, S. *et al.* *Quantitative risk assessment of the effects of climate change on selected causes of death, 2030s and 2050s*. (2014).

3. Carleton, T. *et al.* Valuing the Global Mortality Consequences of Climate Change Accounting for Adaptation Costs and Benefits. 115.

4. Gasparrini, A. *et al.* Projections of temperature-related excess mortality under climate change scenarios. *The Lancet Planetary Health* **1**, e360–e367 (2017).

5. United Nations Department of Social and Economic Affairs. *World Population Prospects 2019*. (UNITED NATIONS, 2019).

6. Barreca, A., Clay, K., Deschenes, O., Greenstone, M. & Shapiro, J. S. Adapting to Climate Change: The Remarkable Decline in the US Temperature-Mortality Relationship over the Twentieth Century. *Journal of Political Economy* **124**, 105–159 (2016).

7. Chen, K., Vicedo-Cabrera, A. M. & Dubrow, R. Projections of Ambient Temperature-and Air Pollution-Related Mortality Burden Under Combined Climate Change and Population Aging Scenarios: a Review. *Current environmental health reports* 1–13 (2020).

8. Carleton, T., Hsiang, S. M. & Burke, M. Conflict in a changing climate. *The European Physical Journal Special Topics* **225**, 489–511 (2016).

9. Hsiang, S. M., Burke, M. & Miguel, E. Quantifying the Influence of Climate on Human Conflict. *Science* **341**, 1235367 (2013).

10. Hall, R. E. & Jones, C. I. The value of life and the rise in health spending. *The Quarterly Journal of Economics* **122**, 39–72 (2007).

11. Jones, C. I. Life and growth. *Journal of political Economy* **124**, 539–578 (2016).

12. Rosen, S. The value of changes in life expectancy. *J Risk Uncertainty* **1**, 285–304 (1988).

13. Blackorby, C., Bossert, W. & Donaldson, D. J. *Population Issues in Social Choice Theory, Welfare Economics, and Ethics*. (Cambridge University Press, 2005). doi:10.1017/CCOL0521825512.

14. Broome, J. Weighing lives. *OUP Catalogue* (2006).

15. Robinson, L. A., Hammitt, J. K. & O’Keeffe, L. Valuing Mortality Risk Reductions in Global Benefit-Cost Analysis. *J. Benefit Cost Anal.* **10**, 15–50 (2019).

16. Ashenfelter, O. & Greenstone, M. Using Mandated Speed Limits to Measure the Value of a Statistical Life. *Journal of Political Economy* **112**, S226–S267 (2004).

17. Kolstad, C. *et al.* Social, economic and ethical concepts and methods. (2014).

18. Broome, J. *Climate Matters: Ethics in a Warming World (Norton Global Ethics Series)*. (W. W. Norton & Company, 2012).

19. Nordhaus, W. D. Revisiting the social cost of carbon. *PNAS* **114**, 1518–1523 (2017).

20. Parfit, D. *Reasons and Persons*. (Clarendon Press, 1984).

21. Ord, T. *The precipice: existential risk and the future of humanity*. (Hachette Books, 2020).

22. Bostrom, N. Existential risk prevention as global priority. *Global Policy* **4**, 15–31 (2013).

23. Scovronick, N. *et al.* Impact of population growth and population ethics on climate change mitigation policy. *Proceedings of the National Academy of Sciences* **114**, 12338–12343 (2017).

24. Committee on Assessing Approaches to Updating the Social Cost of Carbon, Board on Environmental Change and Society, Division of Behavioral and Social Sciences and Education, & National Academies of Sciences, Engineering, and Medicine. *Valuing Climate Changes: Updating Estimation of the Social Cost of Carbon Dioxide*. (National Academies Press, 2017). doi:10.17226/24651.

25. Stern, N. Economics: Current climate models are grossly misleading. *Nature News* **530**, 407 (2016).

26. Pindyck, R. S. Climate change policy: what do the models tell us? *Journal of Economic Literature* **51**, 860–72 (2013).

27. Heal, G. The Economics of the Climate. *Journal of Economic Literature* **55**, 1046–1063 (2017).

28. Nordhaus, W. & Sztorc, P. DICE 2013R: Introduction and user’s manual. *retrieved November* (2013).

29. Bosello, F., Eboli, F. & Pierfederici, R. Assessing the Economic Impacts of Climate Change - An Updated CGE Point of View. *SSRN Journal* (2012) doi:10.2139/ssrn.2004966.

30. Cline, W. R. The economics of global warming. *Institute for International Economics, Washington, DC* 399 (1992).

31. Dellink, R. *et al.* Consequences of climate change damages for economic growth. (2014).

32. Nordhaus, W. D. Geography and macroeconomics: New data and new findings. *Proceedings of the National Academy of Sciences* **103**, 3510–3517 (2006).

33. Nordhaus, W. D. & Boyer, J. *Warming the world: economic models of global warming*. (MIT Press, 2000).

34. Nations, U. World population prospects: The 2017 revision. *United Nations Econ Soc Aff* (2017).

1. See (^1^) for a definition of systematic research synthesis vs. other research synthesis techniques such as meta-analysis and non-systematic research synthesis. [↑](#footnote-ref-2)
2. A variety of disciplines – especially public health, economics, and medicine – have produced studies of the effect of climate change on human mortality. The research synthesis that created the original DICE-2016 climate-economy damage function (^1^) sought to produce an estimate of economic damages from climate change, and therefore conducted their proposed systematic research synthesis using EconLit, which only queries economics literature. [↑](#footnote-ref-3)
3. Because the DICE-2016 model starts from observed 2015 values and optimizes the emissions rate starting in 2020, DICE-EMR also starts from the same 2015 baseline as DICE-2016 and optimizes the emissions trajectory starting in 2020 so that the DICE-EMR optimal emissions trajectory can be compared with the DICE-2016 optimal emissions trajectory on a like-for-like basis. [↑](#footnote-ref-4)
4. Note that a small proportion of emissions in the DICE-2016 model comes from land use change. This is given exogenously, and it is assumed to fall at a rate of 11.5% every 5 years. It is unaffected by the emissions control rate that is chosen to find the optimal emissions trajectory that maximizes the net present value of social welfare. Therefore, when we say “full decarbonization,” this means that all emissions except for emissions from land use change are abated, since the DICE-2016 model assumes that land use decisions are completely exogenous. [↑](#footnote-ref-5)
5. (^1^) has a typo in that the “Dellink 2012” paper referred to in the research synthesis that projects a -1.1% economic impact at 2.5° C warming actually refers to this paper: (^31^). Thanks to Peter Howard for directing me to this. [↑](#footnote-ref-6)
