# Supplementary material for: The mortality cost of carbon
Source: Nat Commun. 2021 Jul 29;12:4467. doi: 10.1038/s41467-021-24487-w (PMC8322393; doi:10.1038/s41467-021-24487-w)
Supplement: Supplementary file 3 — Reporting Summary [file 41467_2021_24487_MOESM3_ESM.pdf]

nature research

Corresponding author(s): R. Daniel Bressler

Last updated by author(s): Apr 26, 2021

Reporting Summary

Nature Research wishes to improve the reproducibility of the work that we publish. This form provides structure for consistency and transparency in reporting. For further information on Nature Research policies, see our Editorial Policies and the Editorial Policy Checklist.

Statistics

For all statistical analyses, confirm that the following items are present in the figure legend, table legend, main text, or Methods section.

n/a	Confirmed	
☒	☐	The exact sample size (n) for each experimental group/condition, given as a discrete number and unit of measurement
☒	☐	A statement on whether measurements were taken from distinct samples or whether the same sample was measured repeatedly
☒	☐	The statistical test(s) used AND whether they are one- or two-sided<br>*Only common tests should be described solely by name; describe more complex techniques in the Methods section.*
☒	☐	A description of all covariates tested
☒	☐	A description of any assumptions or corrections, such as tests of normality and adjustment for multiple comparisons
☐	☒	A full description of the statistical parameters including central tendency (e.g. means) or other basic estimates (e.g. regression coefficient) AND variation (e.g. standard deviation) or associated estimates of uncertainty (e.g. confidence intervals)
☒	☐	For null hypothesis testing, the test statistic (e.g. F, t, r) with confidence intervals, effect sizes, degrees of freedom and P value noted<br>*Give P values as exact values whenever suitable.*
☒	☐	For Bayesian analysis, information on the choice of priors and Markov chain Monte Carlo settings
☒	☐	For hierarchical and complex designs, identification of the appropriate level for tests and full reporting of outcomes
☒	☐	Estimates of effect sizes (e.g. Cohen's d, Pearson's r), indicating how they were calculated

Our web collection on statistics for biologists contains articles on many of the points above.

Software and code

Policy information about availability of computer code

Data collection	No computer code was used to collect data.
Data analysis	Stata MP-16, Wolfram Mathematica 11.3, Matlab r2020b, Excel for Microsoft 365

For manuscripts utilizing custom algorithms or software that are central to the research but not yet described in published literature, software must be made available to editors and reviewers. We strongly encourage code deposition in a community repository (e.g. GitHub). See the Nature Research guidelines for submitting code & software for further information.

Data

Policy information about availability of data

All manuscripts must include a data availability statement. This statement should provide the following information, where applicable:

- Accession codes, unique identifiers, or web links for publicly available datasets
- A list of figures that have associated raw data
- A description of any restrictions on data availability

The data and code used to produce the analysis described in this paper and its supplementary materials is available in the following repository: https://www.openicpsr.org/openicpsr/workspace?goToPath=/openicpsr/138881&goToLevel=project under a creative commons 4.0 license.

Field-specific reporting

Behavioural & social sciences study design

All studies must disclose on these points even when the disclosure is negative.

Study description	This study extends the DICE-2016 integrated assessment model of climate and the economy to include the peer-reviewed literature on the projected mortality effects of climate change. This was done by conducting a systematic research synthesis of the climate-mortality literature as detailed in the paper. This study revises outputs of the DICE-2016 model including the social cost of carbon and the optimal emissions path. In addition, the study quantifies the marginal mortality impact of emissions on excess deaths without monetizing or discounting these deaths in a metric introduced in this paper called the mortality cost of carbon.
Research sample	100 studies were chosen using the systematic research synthesis methodology described in the text and supplementary materials.
Sampling strategy	100 studies were chosen using the systematic research synthesis methodology described in the text and supplementary materials.
Data collection	Data sources and the data collection process are described in detail in the paper (and its supplementary materials). R. Daniel Bressler carried out and documented the whole data collection procedure.
Timing	This study was worked on continuously between November, 2018 and July, 2020 by R. Daniel Bressler. The dates/versions of various data sources used are referenced in the paper. The systematic research synthesis of the climate-mortality literature was conducted in September, 2019.
Data exclusions	None of the 100 studies selected by the systematic research synthesis were excluded prima facie. Each of the studies were considered by R. Daniel Bressler, and a coding and a brief rationale is given for each study as to why it was included or not included in the creation of the mortality damage function. This is all described in detail in the manuscript, the supplementary materials, and all of the underlying details are given in the replication materials.
Non-participation	The method of systematic research synthesis was used to collect the data for this study and not an experiment, so participation and non-participation are not applicable here.
Randomization	100 studies were chosen using the systematic research synthesis methodology described in the text and supplementary materials. This was not an experiment nor a randomized control trial, and I did not randomly select a sample from a population. The systematic research synthesis is intended to find the most relevant studies suitable for this analysis, and then we use the methodology described in the paper to determine which studies contained projections of climate-related mortality that were suitable to contribute towards creating the mortality damage function.

Reporting for specific materials, systems and methods

We require information from authors about some types of materials, experimental systems and methods used in many studies. Here, indicate whether each material, system or method listed is relevant to your study. If you are not sure if a list item applies to your research, read the appropriate section before selecting a response.

Materials & experimental systems

n/a	Involved in the study	
☒	☐	Antibodies
☒	☐	Eukaryotic cell lines
☒	☐	Palaeontology and archaeology
☒	☐	Animals and other organisms
☒	☐	Human research participants
☒	☐	Clinical data
☒	☐	Dual use research of concern

Methods

n/a	Involved in the study	
☒	☐	ChIP-seq
☒	☐	Flow cytometry
☒	☐	MRI-based neuroimaging
